# Online Diagnostic Assessment in Support of Personalized Teaching and Learning: The eDia System

**DOI:** 10.3389/fpsyg.2019.01522

**Published:** 2019-07-03

**Authors:** Benő Csapó, Gyöngyvér Molnár

**Affiliations:** ^1^ MTA-SZTE Research Group on the Development of Competencies, University of Szeged, Szeged, Hungary; ^2^ Department of Learning and Instruction, University of Szeged, Szeged, Hungary

**Keywords:** technology-based assessment, online assessment, diagnostic assessment, assessment framework, item banking

## Abstract

The aims of this paper are: to provide a comprehensive introduction to eDia, an online diagnostic assessment system; to show how the use of technology can contribute to solve certain crucial problems in education by supporting the personalization of learning; and to offer a general reference for further eDia-based studies. The primary function for which the system is designed is to provide regular diagnostic feedback in three main domains of education, reading, mathematics, and science, from the beginning of schooling to the end of the 6 years of primary education. The cognitive foundations of the system, the assessment frameworks, are based on a three-dimensional approach in each domain, distinguishing the psychological (reasoning), the application, and the disciplinary (curricular content) dimensions of learning. The frameworks have been carefully mapped into item banks containing over a 1,000 innovative (multimedia-supported) items in each dimension. The online assessments were piloted, and the system has been operating in experimental mode in over 1,000 schools for several years. This paper outlines the theoretical foundations of the eDia system and summarizes how results from research on the cognitive sciences, learning and instruction, and technology-based assessment have been integrated into a working system designed to assess a large population of students. The paper describes the main functions of eDia and discusses how it supports item writing, constructing tests, online test delivery, automated scoring, data processing, scaling and the provision of feedback both for students and teachers. It shows how diagnostic assessments can be implemented in school practice to facilitate differentiated instruction through regular measurements and to provide instruments for teachers to make formative assessments. Beyond its main function (supporting development toward personalizing education), the eDia platform has been used for assessments in a number of areas from pre-school to higher education both in Hungary and in a number of other countries as well. The paper also reviews results from eDia-based studies and highlights how technology-based assessment extends the possibilities of educational research by making more constructs measurable.

## Introduction

The eDia online assessment system has been built and developed by the Centre for Research on Learning and Instruction, University of Szeged. The principal function for which the system is designed is to provide regular diagnostic information in three main domains of education, reading, mathematics, and science, from the beginning of schooling to the end of the 6 years of primary education. In its present form, the eDia system is an integrated assessment system that is based on sophisticated frameworks and supports assessment processes from item development through test administration and data analyses to well-interpretable feedback. It is one realization of the “integrated, learning-centered assessment systems” envisioned by Pellegrino and Quellmalz (2010).

One of the main challenges of school education stems from the fact that students are different. Looking at the problem from a historical perspective, two main approaches may be identified as school systems have attempted to respond to this challenge: (1) selecting students (ability grouping, tracking, etc.) in the hope that homogeneous classrooms can be set up and (2) accepting different students for heterogeneous classrooms, then differentiating instruction to adjust teaching to the different individual needs of the students (personalization, individualization, etc.). The first option has failed, mostly for two reasons: (1) students are different not only in one dimension but also in a number of different ways, with the differences changing dynamically over time; therefore, (2) the intention of selection has generally resulted in social selection (segregation) with numerous negative side effects. The second option is more promising, and a number of progressive initiatives have emerged in recent decades. However, there have also been a great many difficulties that have stood in the way of personalizing learning; among these, the most prominent is continuously identifying the critical differences between students, differences that determine successful learning options. The most crucial issue in teaching a heterogeneous classroom is teaching students with temporary or permanent difficulties in learning, thus requiring that the difficulties that block their progress be identified.

From a cognitive point of view, the core of the problem was best conceptualized by Ausubel in his frequently cited observation: “The most important single factor influencing learning is what the learner already knows. Ascertain this and teach him accordingly” (Ausubel, 1968, p. vi). As simple as this idea is, it is equally as difficult to implement in heterogeneous classrooms. To realize this in practice, teachers should know “what the learner already knows.” The problem of “knowing what students know,” as has been formulated by several authors (Pellegrino et al., 2001; Opfer et al., 2012), has been solved in general, but making this knowledge useable in practice, teachers should know in “real time,” or at least should receive feedback with sufficient frequency to be able to adjust teaching to the knowledge currently possessed by learners. It is clear that due to material costs and human resources requirements, systematic large-scale diagnostic assessments cannot be conducted with traditional instruments.

In this paper, we first outline the theoretical foundations of the eDia system, including the role of diagnostic assessment, the content of assessment, and the ways to use feedback. Then, we introduce the eDia system, describe its structure, and highlight how technology serves its functions. Finally, we review research studies that have been carried out using eDia.

Throughout this paper, we emphasize that there are a number of innovations that technology brings into numerous aspects of instructional processes, including assessment. However, currently, there is still unexploited potential in the use of technology, including the possibilities of personalizing learning, adjusting teaching and learning processes to the individual needs of students. From a cognitive point of view, if students are always taught what they are prepared for (as Vygotsky’s theory of the zone of proximal development proposes), then they will better comprehend and master the teaching material. From an affective perspective, if each student individually always faces an optimally challenging learning task (as Csíkszentmihályi’s theory of optimal experiences proposes, see Csíkszentmihályi, 2000), both boredom and anxiety are eliminated from learning processes and maintains motivation. The optimal level of challenge supports students’ need for competence, which has a positive impact on students’ intrinsic motivation as well (Ryan and Deci, 2000a,b). We notice here that large item banks also allow personalization of assessment so that each student receives tests adjusted to their actual developmental level (adaptive testing), thus reducing anxiety in the assessment process as well. Both cognitive and affective demands require regular, personalized feedback, which is what eDia is designed for.

## Theoretical Framework

The eDia system constitutes the core of a complex, novel educational model which synthesizes a number of progressive initiatives to improve education. It is designed to support learning and development in the first phase of schooling and takes into account certain realities that determine the possibilities of using technologies. We consider three sets of conditions under which problems must be solved.

1. We assume that the role of teachers remains central in the teaching and learning processes. Their personal presence is needed in the classroom, especially in the first year of schooling. Therefore, the technology in the proposed model is not meant to replace the teacher, but to provide diagnostic tools to support their work. With such diagnostic tools, teachers will be empowered to improve their own work by experimenting, modifying the way they teach and assessing the impact, as research-based teacher education (Westbury et al., 2005; Munthe and Rogne, 2015) prepares them for such activities and as required by evidence-based educational practice (Slavin, 2002).
2. The second reality is the large differences between pupils. We assume, based on evidence from numerous analyses that heterogeneous, inclusive schools and classrooms are more efficient, with both quality and equity potentially ensured simultaneously; however, teaching in heterogeneous classes may be more difficult. The major challenge is to adjust instruction to the individual needs of every student. Diagnostic assessment may help, as it provides information on the actual developmental level of each pupil.
3. We assume that regular feedback is essential for learning. A major trend to provide students with proper feedback has been promoted through formative assessments. We agree with its importance, but at the same time, we assume that teachers are not able to observe every major aspect of learning without an objective assessment instrument. Furthermore, traditional paper-based instruments are not suitable for rapid and frequent feedback. Technology-based diagnostic assessments may fill this gap.

Given these conditions, four major research trends offer results for integration and synthesis that serve as a theoretical foundation for a complex online diagnostic assessment system. (1) In research and development, there is a shift from summative to formative assessment, which provides immediate feedback and direct support for learning. (2) Technology-based assessment has shown enormous progress in the past decade, and ICT infrastructure in schools has improved so that assessment can enter into everyday school practice. (3) Progress in cognitive and educational psychology has produced results which have not yet been exploited in practice and which may contribute to a solution for certain crucial problems, especially in the first year of schooling. (4) Finally, a number of promising models for personalizing learning has had limited influence on practice, mostly because of the lack of easy-to-use assessment instruments. Although efforts within this latter (4) trend highlight the need for regular diagnostic feedback and the reformed teaching methods provide adequate educational context for the assessments, in this section, we only deal in detail with the first (1–3) trends as they have determined the development of the eDia system more directly.

### Formative and Diagnostic Assessment

Large-scale international assessment programs (Trends in International Mathematics and Science Studies – TIMSS, Progress in International Reading Literacy Study – PIRLS, and Program for International Student Assessment – PISA) have had an immense impact on the development of educational systems in many different ways and have inspired the introduction or expansion of national assessment programs. These programs have also advanced testing in a number of areas, including framework development, test administration, data analyses, and reporting. This progress has also highlighted some deficiencies in educational assessment from the perspective of practice as well, for example, the long time between test administration and feedback, the limited usefulness of summative test results with regard to personalized intervention, and the lack or limitations of student-level feedback in general. Another source of dissatisfaction with testing has been the way summative tests have been used in certain countries, especially for high-stakes assessments, e.g., for test-based accountability. These types of testing have caused some negative effects, such as teaching for testing and test score inflation (see, e.g., Koretz, 2018), as well as harmful influence on school climate and teacher stress (Saeki et al., 2018).

These deficiencies have lent a new impetus for other directions in the development of educational assessment and shifted the focus of attention from summative to formative assessment (Clarke, 2001, 2005; Ainsworth and Viegut, 2006; Bennett and Gitomer, 2009; Bennett, 2011; Sheard and Chambers, 2014), or assessment for learning, as it is often called (Black et al., 2003; Hattie and Brown, 2007; Heitink et al., 2016), or diagnostic assessment, to use yet another term (Leighton and Gierl, 2007). There are many different ways formative assessment is used in practice, but a common feature of these assessments is that they reflect students’ learning needs, facilitate understanding in a given context and provide students with immediate feedback (Black and Wiliam, 1998a,b; Black et al., 2004; Good, 2011). There is no sharp distinction between formative and diagnostic assessment, nor does a universal definition for diagnostic assessment exist. However, it is usually described as a kind of assessment which focuses on problems, explores possible difficulties, assesses if students are prepared for a learning task, and thus may measure prerequisite knowledge as well. Furthermore, diagnostic assessment is often followed by a kind of “therapy”: compensatory instruction to eliminate obstacles and offer various forms of supportive activities (e.g., in mathematics: Brendefur et al., 2018), which facilitates data-based decision making (e.g., in reading: Filderman et al., 2018).

One typical and most traditional form of formative assessment takes place in the context of classroom interaction, with evaluation based on teachers’ observation and personal judgment. Further forms are evaluations of students’ work and learning artifacts (performances, presentations, essays, worksheets, projects, documents, lab results, etc.). Although there is a need for frequent personal feedback from teachers, the subjective nature has prompted the use of objective instruments; thus, formative tests have been proposed for this purpose. As these tests have been customized and adjusted to contexts and actual needs, they have usually been teacher-made tests of questionable psychometric quality. Formative tests have been used most systematically in personalized models of instruction, but in any case, their production, administration, and scoring have required immense resources. The use of technology has been proposed to solve these problems, to support certain aspects of the assessments (Feng and Heffernan, 2005; Brown et al., 2008; Feng et al., 2009) or to devise comprehensive assessment systems (Perie et al., 2009).

### Evolution of Technology-Based Assessment

Although technology-based assessment (TBA) is almost as old as the computer itself, modern TBA has a much shorter history. Its potential in assessment has been clear for decades, but it has required several initiatives and the development of the infrastructure at schools to fulfill its promise. We review here only a few major projects and programs that have aided in the realization of eDia as well.

The European Union has launched several initiatives to modernize education, including the expansion of educational assessments to new areas with new technologies. The EU’s Joint Research Centre has organized conferences and workshops to collect experience with TBA projects (Scheuermann and Guimarães Pereira, 2008). One such workshop was held in Reykjavik, Iceland, in September–October 2008 with the participation of over 100 experts presenting several parallel developments (Scheuermann and Björnsson, 2009). Among other software, the TAO program (open source software developed by the Centre de Recherche Public Henri Tudor and EMACS, University of Luxembourg) was introduced in several presentations, indicating that it was not only being used in the PISA studies but also in national initiatives as well (Csapó et al., 2009; Haldane, 2009). The MicroDYN approach (Greiff and Funke, 2009), which later became the core of the PISA 2012 problem-solving assessment and which is also implemented in eDia, was also presented at this meeting. In a volume based on the workshop presentations, three chapters summarized the results of the PISA Computer-Based Assessment of Science by authors from the participating countries (Iceland, Korea, and Denmark; see Halldórsson et al., 2009; Lee, 2009; Sørensen and Andersen, 2009). A chapter in the same volume by Kozma (2009) was also published, which was a call for action to assess and teach the 21st-century skills, a manifesto of the program started around that time.

The Assessment and Teaching of 21st-Century Skills (ATC21S) project was located at the intersection of two major trends in research and development: the need to re-define the purpose of education in the new millennium with a greater focus on the skills required in modern societies and to make these skills measurable through TBA. In the first phase of the project, four working groups were formed to define the targeted skills (Binkley et al., 2012) and to explore methodological, psychometric (Wilson et al., 2012), and technological (Csapó et al., 2012) issues, as well as contextual and environmental issues (Scardamalia et al., 2012). The volume that published the results contained a further chapter on the policy frameworks for the assessments (Darling-Hammond, 2012). In the second phase, the project focused on two prominent and closely related 21st-century skills, collaborative problem-solving and learning in digital networks (Griffin and Care, 2015), thus also contributing to the theoretical and empirical foundations for the 2015 PISA collaborative problem-solving assessment.

The PISA assessments have had an impact on the development of TBA in two major ways: (1) they have advanced the technological background and (2) they have tested the preparedness of individual countries for the assessments, identified deficiencies and exercised some pressure to ensure the necessary conditions to make large-scale TBA possible. The application of TBA started in 2006, when Computer-Based Assessment of Science was an optional domain (OECD, 2010). Only three countries completed the assessments (Denmark, Iceland, and Korea), but this provided an impetus for TBA within PISA. In 2009, the assessment of digital reading was an optional domain. Altogether countries participated, making the comparison of achievement in print and digital reading possible and exploring the new information-processing demands of networking and hyperlinking (OECD, 2011).

The 2012 PISA cycle brought a breakthrough in two respects. First, although paper-based tests remained the main delivery method, the TBA version of assessments was offered as an option for reading and mathematics, making the two delivery methods comparable and linking paper-based and TBA achievement (OECD, 2013). Second, in this cycle, dynamic (creative) problem-solving was the fourth, innovative assessment domain; it used simulation and interaction for the first time on PISA (OECD, 2014). This assessment has had a further impact on the development of TBA. The members of the problem-solving expert group continued meeting, invited further researchers in the field, and published an edited volume, which reported a number of further applications of and innovation in TBA (Csapó and Funke, 2017). The computerized solutions devised for the interaction in the assessment of dynamic problem-solving were adapted and further developed; they were used in 2015 for interactive science items (OECD, 2016) and for collaborative problem-solving (OECD, 2017). In 2015, the transition of PISA to TBA was complete, with all the assessments administered by computer.

The projects and programs reviewed here have influenced the development of the eDia system in several ways. PISA re-defined the content to be measured, while ATC21S linked the skills and technology used for assessment and highlighted the importance of framework development. The technology was developed in interaction with the communities running the projects under review; the major forum, beyond several meetings at conferences, was the Szeged Workshop on Educational Evaluation, held annually at the University of Szeged between 2009 and 2016. The programs reviewed here focused on summative testing among older age groups (secondary schools), underscoring the lack of formative assessment and neglecting the needs of younger students, while recent research in education has emphasized both aspects. The experiences gained from the technological realization of these programs (e.g., the item-builder technology) have been transferred to diagnostic assessments, and eDia has extended them with a number of novel solutions (e.g., item banking, a feedback system, visualization, etc.).

Beyond the developments reviewed here, a parallel evolution took place related to computer-aided instruction (Chauhan, 2017) and intelligent tutoring systems (Kulik and Fletcher, 2016) with significant assessment and feedback components (Conejo et al., 2004). The rapid development of online learning has also advanced TBA, including progress in adaptive testing (e.g., Conejo et al., 2004) and most recently in learning analytics (Avella et al., 2016), which broadens the possibilities of assessing students’ learning and forms of feedback. Strategies based on several forms of computer-aided instruction and online learning designed for older students limit the role of teachers and teach students in specific domains (see, e.g., Chi et al., 2010). They open a different route for personalization and only partially overlap with the type of assessment-based differentiation for which the eDia system is devised (as for these differences, see also Scandura, 2017).

### Determining What to Measure: Three-Dimensional Frameworks for Diagnostic Assessments

Previous assessment projects have stressed the importance of defining the content of assessments, and this is even more significant for diagnostic assessments in the early phases of schooling. Diagnosis requires not only a better understanding of the teaching and learning processes but also the cognitive and affective development of pupils as well. Therefore, framework development has been a prominent component in establishing the eDia system. With a brief description of framework development, we demonstrate that only the use of technology (large item banks and assessments tailored to students’ individual needs) has made it a realistic goal to differentiate the special aspects of learning by defining the three dimensions of assessments.

The reading, mathematics, and science frameworks have been based on a three-dimensional model of learning outcomes. This model takes into account the traditions of defining learning objectives (e.g., creating taxonomies, developing curricula and setting standards; see Csapó, 2004, 2010) and recent research findings in fields ranging from cognitive neuroscience (e.g., Ansari and Coch, 2006) through early childhood education (e.g., McLachlan et al., 2018) to research on teaching and learning in the domains assessed.

The most traditional dimension of learning outcomes is mastering the learning material, i.e., subject matter knowledge, represented in textbooks and defined more generally in the school curricula. This type of knowledge is the easiest for teachers to observe. The most frequently assessed and graded dimension, it is termed the *disciplinary dimension* in the diagnostic frameworks. It has been the central part of many curriculum- or textbook-oriented summative assessments as well as of the first international assessment programs. The PISA frameworks have re-defined the conception of valid knowledge and expanded the interpretation of literacy in a parallel form for the three assessment domains (e.g., OECD, 1999, 2003). The same type of knowledge is assessed in the eDia diagnostic system, which is called the *application dimension*. The third dimension focuses on students’ cognitive development, the processes underlying learning, which is called the *psychological dimension* (for the cognitive foundations, see also the CBAL approach, Bennett, 2010). Although PISA also assesses disciplinary knowledge in mathematics and science, it does so through the applications, while the psychological dimension appears in the innovative domain (e.g., complex problem-solving in 2003, creative problem-solving in 2012, and collaborative problem-solving in 2015). The predecessors to TIMSS focused on knowledge defined in the curricula of the participating countries, so the main resource was disciplinary knowledge, while recent frameworks deal with content, application, and reasoning as well (see, e.g., Mullis et al., 2001, 2005) somewhat similar to the eDia framework. None of the large-scale international assessment programs can measure how well disciplinary knowledge defined in the actual curricula is mastered, but it is defined and assessed in the disciplinary dimension of the diagnostic system.

The three-dimensional frameworks for reading (Csapó and Csépe, 2012), mathematics (Csapó and Szendrei, 2011), and science (Csapó and Szabó, 2012) have been developed by experts in the particular domains and dimensions. In the three domains, a total of nine dimensions are distinguished and defined; the theoretical foundation and previous research on each one are presented in a chapter in the framework volumes. There are similarities between mathematics and science, while reading is somewhat different. The theoretical chapters are followed by the detailed frameworks developed for primary school Grades 1–6. The descriptions are illustrated by sample items showing possible computerized, multimedia-supported item formats to assess a particular dimension. These frameworks served as training materials for the item writers, who then carefully mapped the frameworks into assessment items (over 1,500 items per dimension). They were also used to familiarize the teachers who use eDia with the content of the assessment. These items were empirically piloted, and a further set of books was published, one volume for each domain with detailed descriptions of the assessment dimensions and illustrated by a larger number of items taken from the item banks in the eDia system (Csapó et al., 2015a,b,c). These books help prepare teachers to use the system, to interpret the feedback provided by eDia, and to determine the intervention concluded from the assessment results. Sample items presented in these books also demonstrate that assessing certain aspects of learning (especially the psychological dimension) would be difficult (and almost impossible in school practice) without the use of technology.

The validity of the three-dimensional model has already been empirically tested. Based on the data collected *via* the eDia system, confirmatory factor analyses were performed separately in each grade for each domain. The results confirmed that, although there are usually significant correlations between the dimensions, they assess different psychological constructs (Molnár and Csapó, submitted). The psychometric indicators for the assessments (e.g., reliability) are constantly monitored, items with poor parameters are modified or deleted from the system, and new items are added to improve coverage of the content defined in the frameworks. (Results from quality improvement processes will be published elsewhere.)

## The eDia System

The eDia system began being built in April 2007, when researchers at the University of Szeged implemented the TAO open source software (Plichart et al., 2004) on university servers and began to explore possibilities for it in close cooperation with and with the continuous support of the developers of TAO at the Centre de Recherche Public Henri Tudor, University of Luxembourg. Several pilot studies were completed with TAO, as well as a media effect study to compare the paper-and-pencil and online administration of an inductive reasoning test (Csapó et al., 2009). Although the first results were promising, and by that time several TAO modules had been used in the PISA assessments as well, it soon became obvious that TAO had not been designed for the type of diagnostic assessment system the researchers had aimed to build. This led to a decision to develop new software from scratch optimized for the complex requirements of the diagnostic assessments.

The eDia online diagnostic assessment system can be divided into two main parts. One is the hardware infrastructure (a server farm) and the software that operates the system. This has been developed and optimized for diagnostic assessment, e.g., being continuously accessible for the entire Grade 1–6 student population (up to 600,000 students), and for the management of large item banks (with tens of thousands of items). In addition, this infrastructure can also be used for several other assessment purposes. The other part is the main content of the system, the item banks prepared for the diagnostic assessment of reading, mathematics, and science.

The eDia system is functionally ready for the implementation of systematic assessments and has operated in experimental mode since 2015. At present, there are more than 1,000 partner schools (approx. one-third of the primary schools in Hungary), where it is used on a regular basis. It contains over 25,000 items. The software has been continuously developed, with both the number of partner schools and the number of items available in the system growing.

Currently, three different testing procedures are run with eDia. There are central assessments initiated by the assessment center three times in a school year, at the beginning, in the middle, and at the end of the year. These assessments provide data to establish item parameters and normative reference points. There are teacher-initiated assessments which are used for frequent diagnostic assessments adjusted to the needs of a class or of individual students. The teachers may compile tests out of the items available in the item banks for their own assessment activities. Furthermore, there is testing for research in numerous projects using either items from the item banks or specific tests developed for research purposes.

### Structure of the System: Functions to Serve the Needs of Educational Practice

#### Item Writing

The system contains an item builder module that makes the task of item writing as easy as writing multimedia documents. Item developers receive extensive training in the content of the assessment and in test theory and psychometrics, enabling them to master the use of the item builder module easily (Molnár et al., 2015a,b, 2018). Items are written online, with the draft versions of items undergoing several phases of review (content, language, technical fitness, and format) before they are entered into the item pool for empirical testing. A number of tools are available to support item writing, including templates and scoring schemes. Several items can be created for one stimulus or a set of closely related stimuli; these items together form the tasks. The items in a task can be moved (e.g., added to a test) together.

#### Test Editing

In the present mode of operating the system, tests consisting of a number of tasks form the units of the assessment. Tests may be constructed out of the tasks in several ways. Typically, booklets are formed out of the tasks, and then they can be combined variously into tests, for example, to eliminate the position effect or to optimize linking/anchoring options. Tests can be constructed with adaptive testing techniques, i.e., based on the answers given to all previous items or to items present in the last cluster, to minimize the difference between the students’ ability level and the test difficulty level.

#### Online Test Delivery

Students complete the diagnostic tests as part of their school activity using the available school infrastructure. The tests can be done practically from any device equipped with an internet browser, but the items are optimized for keyboard, mouse, and a large screen. For central assessments, there is an approx. two-week window when eDia is open for the actual assessment. Teacher-initiated testing can take place any time teachers find it useful (at this phase, they are not influenced on how frequently they use it). Students have a specific secret assessment identification code to log into the system.

#### Automated Scoring

The eDia system is designed for both automated and human scoring. However, the items in the item banks that are prepared for the regular diagnostic assessments are scored automatically, with human scoring reserved for research and specific applications. Automatic scoring makes it possible to provide immediate feedback, and it is necessary for the rapid scoring of a large number of assessments. The system offers a variety of scoring options, adjusted to item type and form of response capture.

#### Built-In Data Processing and Statistical Analyses

The eDia system contains a statistical analytics module, which can perform every computation required by the assessment from descriptive statistics through classical test theory to IRT modeling. The computations are programmed using the open source “R” programming language and are continuously adapted to the developing system. The data can be exported from the system for further analyses.

#### Teacher-Assembled Tests

Teachers have been encouraged to use objective assessment instruments since the very beginning of educational testing; however, most tests available for classroom assessment are summative tests. Such tests are difficult to adapt to the actual needs of a class, not to mention individual students. Another option is teacher-made tests, but the time and resources needed to prepare and score them hinder practical use. The teacher-assembled tests in eDia fill this gap. Participating teachers are granted access to the item banks, so they can assemble tests out of available tasks. These tests can then be administered to individual students, a group of students or an entire class, with the results made available immediately after testing. Models for the co-existence of centrally initiated tests and teachers’ assessment are under development. The current model is that central assessments serve a screening function, while teacher-initiated tests are mostly used for formative and diagnostic purposes if needed. Further options are being explored, e.g., automated recommendations for testing based on previous assessment results.

#### Feedback

At present, there are two basic forms of feedback. One is the immediate feedback students receive right after the test has been completed in the form of percentage of total score of a particular test. Another form is contextualized information based on normative reference data, available only after the central assessments. After the general assessments, both students and teachers receive detailed information about the results for each assessment dimension. Students may download a PDF file with a detailed description of the content of the assessment and their own achievement compared to the national norm and class mean. Teachers receive similar information on their students individually in each dimension as well as a comprehensive, contextualized picture of their class, comparing it to other members of the same age group in the entire school, school district, region, and country. This feedback is provided in graphic form as well to help teachers comprehend and use the data.

#### Scaling and Setting Norms

An IRT model is used to establish assessment scales. There are nine distinct scales in the eDia system as they are defined in the assessment framework; each one is developed separately. Establishing normative scales is a long process, one which requires several steps in the case of the eDia system. The results of the end-of-year assessments are used to establish the scales. In the first step, separate norms are defined for the different grades, with the mean for a grade set for 500 with a SD of 100. This phase has already been completed, and the 54 (6 grades × 3 domains × 3 dimensions) reference scales have been established.

The next step is to devise developmental scales with vertical scaling of the data, linking the achievement of the different grades. This can be done easily with a psychological dimension, where a more or less continuous development can be assumed. As cognitive development is stimulated by out-of-school experiences as well, there may be large differences within a given cohort; some students’ achievement may be closer to the mean for a different cohort. Thus, linking the grades causes no difficulties. These considerations are only partially appropriate for the application dimensions, while the disciplinary dimensions are based on the material taught. Therefore, students in a particular grade may only be offered tasks from earlier grades, but not from later ones. Due to these complications, the first vertical scales for the psychological dimensions have already been prepared (see Molnár and Csapó, submitted), but vertical scaling in the other two dimensions requires more sophisticated statistical procedures (e.g., multidimensional IRT).

Finally, longitudinal scales will also be devised, making it possible to monitor student progress and to observe how they progress within a given period, compared to his/her previous and others’ mean change. Developing such scales requires even more care and time and is especially difficult because collecting longitudinal data from the period covered by eDia takes at least 5 years, while the social and contextual conditions are also rapidly changing in the meantime. On the other hand, eDia does not provide high-stakes testing, nor is producing trend data a requirement. Thus, it can be flexible in establishing normative scales. Whatever the means used for scaling, scale development should also serve the formative, diagnostic function of the system.

### Novel Item Formats for Improving the Quality of Testing

Quality of testing can be defined in terms of validity (including predictive and diagnostic validity), reliability, and objectivity. In this section, we show how new item formats made possible by technology can improve the quality of testing. A number of media effect studies have been carried out in past decades to explore most aspects of assessments. The quality of TBA is usually compared to paper-and-pencil or face-to-face testing, so we also compare the eDia items to these traditional testing modes. Technology offers numerous new options both in presenting stimuli and in capturing students’ responses that are not possible through traditional testing modes; in addition, technology improves objectivity and validity significantly (for a detailed discussion of technological issues, see Csapó et al., 2012).

#### New Forms of Stimuli

Use of technology expands the possibilities of creating more life-like situations and using more authentic stimuli. There are three ways to develop computer-based tests, tasks, and items. First, tests/tasks/items can be prepared according to traditional approaches with designs based on paper-and-pencil techniques. Texts, static images, schematic figures, and graphs are also available on paper, but their richness and variety represent an added value of TBA. We call these kinds of computer-based tasks first-generation tasks (Molnár et al., 2017). Second-generation tests contain tasks with new formats, including multimedia (e.g., animation, video and audio), constructed response, automatic item generation, and automatic scoring tests (Pachler et al., 2010), thus increasing the level of authenticity and the power of assessment. These types of tasks cannot be administered in paper-and-pencil format. Finally, third-generation tests dramatically increase the level of reality and the number of ways students can demonstrate their skills as they allow students to interact with complex scenarios (e.g., complex problem-solving items in the MicroDYN approach), simulations (html documents to imitate a closed internet environment), situations (e.g., GeoGebra elements), and dynamically changing items and/or to collaborate online with other students to solve dynamically changing, interactive problem-solving items. All of these options are implemented and available for item development in the eDia system.

Any kind of multimedia, animation, video, voice, etc. provides authentic content, improves validity, and serves specific functions. Special accommodations can be embedded into technology-based tests; for example, validity of test results can be enhanced by providing instructions both in an on-screen written form and with a pre-recorded voice, thereby preventing failures caused by students’ reading difficulties. Thus, in the eDia system, students in Grades 1–3 can listen to instructions on headphones while the tests are being administered. It is also possible to standardize the test environment by controlling the presentation of information in different ways (e.g., timing and a given number of repetitions).

#### New Forms for Response Capture

Use of technology changes not only the forms of stimuli but also those of response capture. In the traditional test environment, response capture happened basically by circling, ticking, X-ing, underlining or writing letters, numbers, words or sentences. The TBA environment expands these options, but this expansion strongly depends on the technology used. There are different possibilities for response capture in the case of a tablet or a desktop computer. The eDia system is prepared for both. However, as the keyboard and mouse are used for input in most Hungarian schools, the eDia task responses are optimized for them.

The TBA environment makes it possible to expand the possibilities of manipulation with task elements and to realize the following forms of response capture with a mouse: (1) clicking on form elements (radio button and checkbox), (2) using a drop-down menu, (3) clicking on pictures or parts of pictures, (4) clicking on texts or parts of texts, (5) coloring shapes or pictures or parts of them by clicking, (6) sequencing by ordering mouse clicks, (7) connecting two task elements with lines or arrows, (8) constructing answers with on-screen manipulations with drag-and-drop letters, words, sentences, numbers, shapes, pictures, voices, sounds, animations, simulations, etc., that is, all kinds of task elements, and (9) using sliders and functions or other changeable and interactive task elements. Other possibilities are available with the keyboard, such as typing letters, numbers, and words. Logging and analyzing log data by measuring response time, mouse movement, and navigation sequence to describe the activity of the students during testing can also contribute to more elaborated feedback; however, further studies are required to explore how to use these methods more effectively. All these possibilities for logging students’ activities while they respond to items are available in the eDia system.

#### Complex Item Formats: Interactivity and Simulation

The eDia system was prepared to administer third-generation tests. The MicroDYN-based assessment of problem-solving (Greiff and Funke, 2009; Greiff et al., 2013; Molnár and Csapó, 2018) is available with a large number of items. One of the benefits of MicroDYN is that it allows various independent and dependent variables, and different connections may be defined between them for the simulated systems. The difficulty level of the task may thus easily be changed. A further expansion of this conception is the assessment of collaborative problem-solving. It makes it possible to use a real human-human scenario during data collection (Pásztor-Kovács et al., 2018). This allows more social interaction, compared to the PISA 2015 collaborative problem-solving assessment, which used human-agent interaction (OECD, 2017). Further simulation-based items were used on an ICT literacy test (Tongori, 2018). These complex item formats have been used for assessments beyond the diagnostic system and for experimentation and research, and these experiences will also be applied to the diagnostic assessments.

## Beyond Diagnostic Assessment: eDia As A Research Instrument

Beyond its main purpose of providing diagnostic assessments, the eDia platform has been used in a number of other domains and in research projects as well. In this section, we review the research in which data were collected by eDia.

### Further Assessment Domains Implemented in eDia

At present, there are over 20 further domains (called minor domains) for which tests or test batteries are implemented on the eDia platform. The principle in general is that different tests are prepared for the different age groups linked with anchor items.

Supporting the kindergarten-school transition with assessment instruments is one of the current extensions of the eDia. First, the DIFER test battery, a broadly used face-to-face instrument, was digitized, and then the traditional and online delivery methods were compared. Results from the media effect study indicated that the two versions (face-to-face vs. online) were equivalent and that the digitized version was not only more convenient to use, but the objectivity and reliability had also improved on some subtests (Csapó et al., 2014). Based on these experiences, a new school readiness test battery has been developed and optimized for online assessment, which can be used in kindergarten with tablets (Csapó et al., 2017, 2018).

Several instruments were devised for assessments of curricular areas beyond the three major domains. The media effect on composing skills was studied with primary school students (Nagy, 2015). A test of musical abilities used pre-recorded sound stimuli for melody and rhythm (Asztalos and Csapó, 2017). Several tests were prepared for English and German as a Second Language (reading, listening, and vocabulary), while the TBA made it possible to use authentic voice recordings to assess listening skills (Vígh et al., 2015; Nikolov and Csapó, 2017, 2018; Habók and Magyar, 2018a, 2019). Assessments of visual skills benefitted especially from the possibilities of rich illustrations (Kárpáti et al., 2015). Online tests have also been prepared for cross-curricular competencies, such as learning to learn (Habók, 2015; Vainikainen et al., 2015), health literacy (Nagy et al., 2015), financial literacy (Tóth, 2015), ICT literacy (Molnár et al., 2015b), and civic competencies (Kinyó, 2015).

Assessment of a variety of reasoning skills is embedded in the mathematics and science psychology dimension, mostly operational reasoning skills. However, there are some skills that play a distinct role in learning and cognitive development; therefore, comprehensive instruments have been prepared to assess them. Inductive reasoning is one of the most frequently assessed higher-order thinking skills, and several inductive reasoning tests have been developed for the eDia as well. First, a widely used paper-and-pencil inductive reasoning test (verbal and numerical analogies, and number series, see Csapó, 1997) was migrated to the digital platform (Csapó et al., 2009). Later, other tests based on Klauer’s model (see, e.g., Klauer and Phye, 2008) were prepared (Molnár et al., 2013) and used in a number of national and international projects. Specific item formats were developed to assess dynamic problem-solving (the MicryDYN base, see Molnár and Pásztor-Kovács, 2015; Csapó and Molnár, 2017a), collaborative problem-solving (e.g., interactivity and communicating with pre-defined messages, see Pásztor-Kovács et al., 2018), creativity (divergent thinking and a program for counting rare solutions, see Pásztor et al., 2015), and combinatorial reasoning (drag-and-drop to combine elements and an algorithm to distinguish valid and invalid combinations, see Pásztor et al., 2015).

Tests, test batteries, and questionnaires beyond the cognitive domain are also implemented through eDia. Some of them are essential for successful learning, but because of the lack of easy-to-use instruments, they are rarely assessed. Motivation is one such affective attribute, and a related mastery motivation questionnaire is available on eDia (Józsa et al., 2015; Zsolnai and Kasik, 2015), as well as a self-regulated foreign language learning strategy questionnaire (Habók and Magyar, 2018b). The PISA 2020 learning strategy questionnaire (Artelt et al., 2003) has also been implemented and used in several projects (e.g., Csapó and Molnár, 2017a). Experimenting with the assessment of further affective and social skills is also in progress (e.g., Zsolnai and Kasik, 2015).

The eDia platform has been used in higher education. For example, in 2015, the University of Szeged introduced an assessment system to explore how well incoming students are prepared for university studies. In the first year, six tests were administered through eDia: Hungarian language and literature (with a strong reading comprehension component), mathematics, history, science and English as a foreign language as well as a dynamic problem-solving test (Csapó and Molnár, 2017a). Since then, the system has evolved further (Molnár and Csapó, 2019b).

### Applications of eDia in International Assessments; Comparative Studies

The eDia system has been used for research within international collaborative projects carried out by the University of Szeged Centre for Research on Learning and Instruction and supports investigations by PhD students at the Doctoral School of Education at the same university. In this section, we review some results of these efforts, highlighting new opportunities for educational research offered by the online assessment.

In Finland, the Centre for Educational Assessment, University of Helsinki, cooperates with Vantaa city schools in using tablets in everyday teaching and learning processes. Within the framework of this project, Hungarian tests were translated into Finnish and assessments were carried out in both countries using the same instruments, with the tests delivered from the University of Szeged servers (Hotulainen et al., 2018; Pásztor et al., 2018). The first results may indicate the impact of frequent testing, but further studies would be required to uncover the mechanisms.

The tests for assessing thinking skills implemented in the eDia have been used in several international studies. The knowledge acquisition phase of dynamic problem-solving involves two further skills, combinatorial reasoning (systematically combining possible values of independent variables) and inductive reasoning (rule induction and generalizing the experience of interactions). The relationships of these skills were explored; the dynamic problem-solving tests, together with combinatorial and inductive reasoning tests were translated into Chinese and were administered to Chinese students. The results indicated a stronger impact of combinatorial reasoning than that of inductive reasoning (Wu and Molnár, 2018a). The relationship between problem-solving, creativity, inductive reasoning, and working memory was explored in a similar study (Wu and Molnár, 2018b). In Namibia, the relationship between scientific reasoning and motivation to learn science was examined (Kambeyo et al., 2017) as well as the possibilities of online assessment of scientific inquiry skills. These studies indicated that online assessment is feasible even with a modest school infrastructure.

Another set of studies was completed on learning foreign languages in three countries, Mongolia (Ragchaa, 2017), Kazakhstan (Akhmetova and Csapó, 2018), and Azerbaijan (Karimova and Csapó, 2018), where the two most frequently studied foreign languages are English and Russian. Thus, these countries offer different contexts and sets of conditions than those of Hungary, where the main foreign languages are English and German (see, e.g., Nikolov and Csapó, 2018). Another difference is that these countries use the Cyrillic alphabet. Several research questions were explored in these studies on learning foreign languages with eDia-based instruments, including the development of receptive skills, self-concept and learning strategies.

### Assessment Platform for the Hungarian Educational Longitudinal Program

The Hungarian Educational Longitudinal Program (HELP) was launched in 2003 and is maintained by the SZTE-MTA Research Group on the Development of Competencies (Csapó, 2007). A new cohort (a nationally representative sample of approx. 6,000 students) is added to the program every 4 years, with students being monitored from the beginning of schooling to the end of compulsory education. Data collection has focused on three main domains, reading, mathematics, and science, and data are systematically collected on a number of cognitive, affective, and contextual variables. The online assessment has been gradually introduced to the data collection effort (e.g., languages have been tested online, see Nikolov and Csapó, 2018), with the cohort that entered school in 2015 having been exclusively assessed with the eDia instruments. The benefit of longitudinal research from the perspective of developing the diagnostic system is that it offers a nationally representative sample for scale development and for determining the predictive power of certain instruments (e.g., school readiness tests, see Csapó et al., 2018).

## Discussion And Conclusions

### Practical Relevance and Limitations of the Online Assessment

Systematic feedback is a basic condition for the operation and development of any complex system and providing students and teachers with an inexpensive, easy-to-use, valid, and reliable assessment system may significantly contribute to solving certain crucial problems of education today. Making it possible to measure the different dimensions of learning separately, especially the mostly hidden psychological dimension, i.e., thinking and cognitive development may support meaningful learning and a deeper conceptual understanding. (Empirical studies concerning these assumptions are in progress; see also Molnár and Csapó, 2019a).

Teachers see the differences between their students and realize if some of their students fail, but without proper instruments teachers cannot determine the nature and magnitude of the differences with precision. Diagnostic assessments support the personalization of learning, adjusting teaching to students’ personal needs. Teachers routinely use certain types of formative assessment (mostly based on their subjective observation), and we may assume that with better instruments they will teach better. However, we may not assume that they will be able to fully exploit the potential of online diagnostic assessments; they need training to empower them. Several training programs (from one-day introductory workshops to two-year training of assessment experts) are available within the framework of the project. Ideally, the teacher-training component is an in-service adaptation of research-based teacher education (see, e.g., Munthe and Rogne, 2015).

As there is a growing concern among teachers about high-stakes testing and the use of its results for accountability (Tóth, 2011), monitoring their views on diagnostic assessment will be an important task. An indicator of acceptance of eDia is that teachers and schools have been participating in the assessments voluntarily, with informal communication confirming its acceptance as well. Formal surveys will be needed to gain a better understanding of teachers’ opinions.

Finally, we have to emphasize that an assessment instrument alone does not improve the quality of learning; its practical impact depends on how the information it provides is used to change teaching and learning processes. To better use the power of feedback, the conception of classroom teaching should basically be changed; there is a need for new models of teaching and learning, where students’ individual needs are better served. Such models have existed for decades, but the lack of appropriate tools has hindered large-scale use. In the most general terms, Mastery Learning is one such model, which, supported with online pre-tests and post-tests, may gain a new impetus (Csapó and Molnár, 2017b). There are also several promising new models which stress the role of regular feedback and use of assessment data made possible by TBA, e.g., data-based teaching (Datnow and Hubbard, 2016) and assessment-powered teaching (Sindelar, 2010). Experience in the areas of computer aided-instruction and tutoring systems (Kulik and Fletcher, 2016; Chauhan, 2017) may be used, especially in stimulating students’ development in the psychological dimensions when diagnostic assessments indicate the need for such intervention.

### Further Research Prospects

Regular diagnostic assessments generate large databases and render it possible to make further sophisticated use of those that have already been started in other areas (see research on the “data revolution” and “big data”). Educational data mining and process mining have already produced results applicable in practice as well (Tóth et al., 2017). Certain methods developed within the paradigm of learning analytics may also be used to process databases produced by diagnostic assessments as well.

Log file analysis is the easiest and most appropriate new method for using new types of assessment data (metadata and log data). An easily recordable and already routinely used piece of information is the time students spend on certain activities when completing online tasks; time-on-task analyses, among other methods, may indicate students’ attention and motivation. Some item types (combinatorial reasoning task enumerations, MicroDYN items and collaborative problem-solving activities) allow the recording of more detailed information on students’ reasoning. Some analyses (e.g., latent class analyses) using data collected with eDia have already been conducted (Greiff et al., 2018; Molnár and Csapó, 2018), but further research is needed to find ways to make practical use of these results, adding new analytical modules to the eDia platform, creating new, log data-based indicators and supporting students’ cognitive development in the long run.

## Author Contributions

Both of the authors, BC and GM, certify that they have participated sufficiently in the study to take responsibility for the content, including writing and final approval of the manuscript. Each author agrees to be accountable for all aspects of the paper.

### Conflict of Interest Statement

The authors declare that the research was conducted in the absence of any commercial or financial relationships that could be construed as a potential conflict of interest.

## References

[ref1] Ainsworth L. Viegut D. (2006). Common formative assessments. How to connect standards-based instruction and assessment. (Thousand Oaks, CA: Corwin Press).

[ref2] Akhmetova A. Csapó B. (2018). Development of reading skills of 6th and 8th graders in English, Kazakh, and Russian from the perspective of young learners’ backgrounds in Pavlodar. *XVIII. Országos Neveléstudományi Konferencia*; Budapest.

[ref3] Ansari D. Coch D. (2006). Bridges over troubled water: education and cognitive neuroscience. Trends Cogn. Sci. 10, 146–151. 10.1016/j.tics.2006.02.007, PMID: 16530462

[ref4] Artelt C. Baumert J. Julius-McElvani N. Peschar J. (2003). Learners for life. Student approaches to learning. Results from PISA 2000. (Paris: OECD).

[ref5] Asztalos K. Csapó B. (2017). Development of musical abilities: cross-sectional computer-based assessments in educational contexts. Psychol. Music 45, 682–698. 10.1177/0305735616678055

[ref6] Ausubel D. P. (1968). Educational psychology: A cognitive view. (New York: Holt, Rinehart and Winston).

[ref7] Avella J. T. Kebritchi M. Nunn S. G. Kanai T. (2016). Learning analytics methods, benefits, and challenges in higher education: a systematic literature review. Online Learn. 20, 13–29.

[ref8] Bennett R. E. (2010). Cognitively based assessment of, for, and as learning (CBAL): a preliminary theory of action for summative and formative assessment. Measurement 8, 70–91. 10.1080/15366367.2010.508686

[ref9] Bennett R. E. (2011). Formative assessment: a critical review. Assess. Educ. Princ. Policy Pract. 18, 5–25. 10.1080/0969594x.2010.513678

[ref10] Bennett R. E. Gitomer D. H. (2009). “Transforming K-12 assessment” in Assessment issues of the 21st century. eds. Wyatt-Smith C. Cumming J. (New York, NY: Springer), 43–61.

[ref11] Binkley M. Erstad O. Herman J. Raizen S. Ripley M. Miller-Ricci M. (2012). “Defining twenty-first century skills” in Assessment and teaching of 21st century skills. eds. Griffin P. McGaw B. Care E. (New York: Springer), 17–66.

[ref13] Black P. Harrison C. Lee C. Marshall B. William D. (2003). Assessment for learning. Putting it into practice. (Berkshire: Open University Press).

[ref12] Black P. Harrison C. Lee C. Marshall B. Wiliam D. (2004). Working inside the black box: assessment for learning in the classroom. Phi Delta Kappan 86, 8–21. 10.1177/003172170408600105

[ref14] Black P. Wiliam D. (1998a). Inside the black box: Raising standards through classroom assessment. (London, UK: King’s College).

[ref15] Black P. Wiliam D. (1998b). Assessment and classroom learning. Assess. Educ. Princ. Policy Pract. 5, 7–74. 10.1080/0969595980050102

[ref16] Brendefur J. L. Johnson E. S. Thiede K. W. Strother S. Severson H. H. (2018). Developing a multi-dimensional early elementary mathematics screener and diagnostic tool: the primary mathematics assessment. Early Childhood Educ. J. 46, 153–157. 10.1007/s10643-017-0854-x, PMID: 29576730 PMC5859347

[ref17] Brown J. Hinze S. Pellegrino J. W. (2008). “Technology and formative assessment” in 21st century education. Vol 2. Technology. ed. Good T. (Thousand Oaks, CA: Sage), 245–255.

[ref18] Chauhan S. (2017). A meta-analysis of the impact of technology on learning effectiveness of elementary students. Comput. Educ. 105, 14–30. 10.1016/j.compedu.2016.11.005

[ref19] Chi M. VanLehn K. Litman D. Jordan P. (2010). “Inducing effective pedagogical strategies using learning context features” in User modeling, adaptation and personalization: 18th international conference, UMAP 2010. eds. De Bra P. Kobsa A. Chin D. (Heidelberg: Springer), 147–158.

[ref20] Clarke S. (2001). Unlocking formative assessment. Practical strategies for enhancing pupils learning in primary classroom. (London: Hodder Arnold).

[ref21] Clarke S. (2005). Formative assessment in action. Weaving the elements together. (London: Hodder Murray).

[ref22] Conejo R. Guzmán E. Millán E. Trella M. Pérez-De-La-Cruz J. L. Ríos A. (2004). SIETTE: a web-based tool for adaptive testing. Int. J. Artif. Intell. Educ. 14, 29–61.

[ref23] Conejo R. Millán E. Pérez-de-la-Cruz J. L. Trella M. (2000). “An empirical approach to on-line learning in SIETTE” in Intelligent tutoring systems. Proceedings of the 5th international conference on intelligent tutoring systems. eds. Gauthier G. Frasson C. VanLehn K. (Berlin, Heidelberg: Springer), 605–614.

[ref24] Csapó B. (1997). The development of inductive reasoning: cross-sectional measurements in an educational context. Int. J. Behav. Dev. 20, 609–626.

[ref25] Csapó B. (2004). “Knowledge and competencies” in The integrated person. How curriculum development relates to new competencies. ed. Letschert J. (CIDREE: Enschede), 35–49.

[ref26] Csapó B. (2007). Hosszmetszeti felmérések iskolai kontextusban – az első átfogó magyar iskolai longitudinális kutatási program elméleti és módszertani keretei [Longitudinal assessments in school context – theoretical and methodological frames of the first large-scale school-related longitudinal program in Hungary]. Magyar Pedagógia 107, 321–355.

[ref27] Csapó B. (2010). Goals of learning and the organization of knowledge. Z. Pädagog. 2010(Suppl. 56), 12–27.

[ref28] Csapó B. Ainley J. Bennett R. Latour T. Law N. (2012). “Technological issues of computer-based assessment of 21st century skills” in Assessment and teaching of 21st century skills. eds. Griffin P. McGaw B. Care E. (New York: Springer), 143–230.

[ref29] Csapó B. Csépe V. (eds.) (2012). Framework for diagnostic assessment of reading. (Budapest: Nemzeti Tankönyvkiadó).

[ref30] Csapó B. Csíkos C. Molnár G. (eds.) (2015a). A matematikai tudás diagnosztikus értékelésének tartalmi keretei [Framework for online diagnostic assessment of mathematics knowledge]. (Budapest: Oktatáskutató és Fejlesztő Intézet).

[ref31] Csapó B. Funke J. (eds.) (2017). The nature of problem solving. Using research to inspire 21st century learning. (Paris: OECD).

[ref32] Csapó B. Hódi Á. Kiss R. Pásztor A. Rausch A. Molnár G. (2017). Developing online diagnostic instruments for assessing pupils’ skills at the beginning of schooling. *Paper presented at the 17th biennial conference for research on learning and instruction*; Tampere.

[ref33] Csapó B. Korom E. Molnár G. (eds.) (2015b). A természettudományi tudás diagnosztikus értékelésének tartalmi keretei [Framework for online diagnostic assessment of science knowledge]. (Budapest: Oktatáskutató és Fejlesztő Intézet).

[ref34] Csapó B. Molnár G. (2017a). Potential for assessing dynamic problem-solving at the beginning of higher education studies. Front. Psychol. 8, 1–12. 10.3389/fpsyg.2017.02022 29209255 PMC5701914

[ref35] Csapó B. Molnár G. (2017b). “Assessment-based, personalized learning in primary education” in Knowledge management in the 21st century: Resilience, creativity and co-creation. Proceedings of IFKAD 2017. eds. Spender J. C. Schiuma G. Gavrilova T. (St. Petersburg: St. Petersburg University Graduate School of Management), 443–449.

[ref36] Csapó B. Molnár G. Nagy J. (2014). Computer-based assessment of school-readiness and reasoning skills. J. Educ. Psychol. 106, 639–650. 10.1037/a0035756

[ref37] Csapó B. Molnár G. Pásztor A (2018). Predictive validity of technology-based school-readiness assessments. Paper presented at the 9th biennial conference of EARLI SIG 1, assessment and evaluation: Assessment & learning analytics; Helsinki.

[ref38] Csapó B. Molnár G. Tóth R. K. (2009). “Comparing paper-and-pencil and online assessment of reasoning skills. A pilot study for introducing electronic testing in large-scale assessment in Hungary” in The transition to computer-based assessment. New approaches to skills assessment and implications for large-scale testing. eds. Scheuermann F. Björnsson J. (Luxemburg: Office for Official Publications of the European Communities), 120–125.

[ref39] Csapó B. Pásztor A. (2015). “A kombinatív képesség fejlődésének mérése online tesztekkel [Assessment of the development of combinative ability with online tests]” in Online diagnosztikus mérések az iskola kezdő szakaszában [Online diagnostic assessments in the beginning phase of schooling]. eds. Csapó B. Zsolnai A. (Budapest: Oktatáskutató és Fejlesztő Intézet), 367–386.

[ref40] Csapó B. Steklács J. Molnár G. (eds.) (2015c). Az olvasás-szövegértés online diagnosztikus értékelésének tartalmi keretei [Framework for online diagnostic assessment of reading comprehension]. (Budapest: Oktatáskutató és Fejlesztő Intézet).

[ref41] Csapó B. Szabó G. (eds.) (2012). Framework for diagnostic assessment of science. (Budapest: Nemzeti Tankönyvkiadó).

[ref42] Csapó B. Szendrei M. (eds.) (2011). Framework for diagnostic assessment of mathematics. (Budapest: Nemzeti Tankönyvkiadó).

[ref43] Csíkszentmihályi M. (2000). Beyond boredom and anxiety. (San Francisco: Jossey-Bass). (Original work published 1975).

[ref44] Darling-Hammond D. (2012). “Policy frameworks for new assessments” in Assessment and teaching of 21st century skills. eds. Griffin P. McGaw B. Care E. (New York: Springer), 301–339.

[ref45] Datnow A. Hubbard L. (2016). Teacher capacity for and beliefs about data-driven decision making: a literature review of international research. J. Educ. Chang. 17, 7–28. 10.1007/s10833-015-9264-2

[ref46] Farcot M. Latour T. (2009). “Transitioning to computer-based assessments: a question of costs” in The transition to computer-based assessment. New approaches to skills assessment and implications for large-scale testing. eds. Scheuermann F. Björnsson J. (Luxemburg: Office for Official Publications of the European Communities), 108–116.

[ref47] Feng M. Heffernan N. T. (2005). Informing teachers live about student learning: reporting in the assistment system. Technol. Instr. Cogn. Learn. 3, 1–14.

[ref48] Feng M. Heffernan N. T. Koedinger K. R. (2009). Addressing the assessment challenge in an online system that tutors as it assesses. User Model. User-Adapt. Interact. 19, 243–266. 10.1007/s11257-009-9063-7

[ref49] Filderman M. J. Toste J. R. Didion L. A. Peng P. Clemens N. H. (2018). Data-based decision making in reading interventions: a synthesis and meta-analysis of the effects for struggling readers. J. Spec. Educ. 52, 174–187. 10.1177/0022466918790001

[ref50] Good R. (2011). Formative use of assessment information: it’s a process, so let’s say what we mean. Pract. Assess. Res. Eval. 16, 1–6.

[ref51] Greiff S. Funke J. (2009). “Measuring complex problem solving: the MicroDYN approach” in The transition to computer-based assessment. New approaches to skills assessment and implications for large-scale testing. eds. Scheuermann F. Björnsson J. (Luxemburg: Office for Official Publications of the European Communities), 157–163.

[ref52] Greiff S. Molnár G. Martin R. Zimmermann J. Csapó B. (2018). Students’ exploration strategies in computer-simulated complex problem environments: a latent class approach. Comput. Educ. 126, 248–263. 10.1016/j.compedu.2018.07.013

[ref53] Greiff S. Wüstenberg S. Holt D. V. Goldhammer F. Funke J. (2013). Computer-based assessment of complex problem solving: concept, implementation, and application. Educ. Technol. Res. Dev. 61, 407–421. 10.1007/s11423-013-9301-x

[ref54] Griffin P. Care E. (eds.) (2015). Assessment and teaching of 21st century skills: Methods and approach. (New York: Springer).

[ref55] Habók A. (2015). “A tanulás tanulásának vizsgálata online környezetben [Exploration of learning to learn in an online environment]” in Online diagnosztikus mérések az iskola kezdő szakaszában [Online diagnostic assessments in the beginning phase of schooling]. eds. Csapó B. Zsolnai A. (Budapest: Oktatáskutató és Fejlesztő Intézet), 179–198.

[ref56] Habók A. Magyar A. (2018a). The effect of language learning strategies on proficiency, attitudes and school achievement. Front. Psychol. 8:2358. 10.3389/fpsyg.2017.02358 29379461 PMC5770794

[ref57] Habók A. Magyar A. (2018b). Validation of a self-regulated foreign language learning strategy questionnaire through multidimensional modelling. Front. Psychol. 9:1388. 10.3389/fpsyg.2018.01388 30127759 PMC6087767

[ref58] Habók A. Magyar A. (2019). The effects of EFL reading comprehension and certain learning related factors on EFL learners’ reading strategy use. Cogent Educ. 6, 1–19. 10.1080/2331186x.2019.1616522

[ref59] Haldane S. (2009). “Delivery platforms for national and international computer-based surveys: history, issues and current status” in The transition to computer-based assessment. New approaches to skills assessment and implications for large-scale testing. eds. Scheuermann F. Björnsson J. (Luxemburg: Office for Official Publications of the European Communities), 63–67.

[ref60] Halldórsson A. M. McKelvie P. Björnsson J. K. (2009). “Are Icelandic boys really better on computerized tests than conventional ones? Interaction between gender, test modality and test performance” in The transition to computer-based assessment. New approaches to skills assessment and implications for large-scale testing. eds. Scheuermann F. Björnsson J. (Luxemburg: Office for Official Publications of the European Communities), 178–193.

[ref61] Hattie J. A. Brown G. T. (2007). Technology for school-based assessment and assessment for learning: development principles from New Zealand. J. Educ. Technol. Syst. 36, 189–201. 10.2190/et.36.2.g

[ref62] Heitink M. C. Van der Kleij F. M. Veldkamp B. P. Schildkamp K. Kippers W. B. (2016). A systematic review of prerequisites for implementing assessment for learning in classroom practice. Educ. Res. Rev. 17, 50–62. 10.1016/j.edurev.2015.12.002

[ref63] Hotulainen R. Pásztor A. Kupiainen S. Molnár G. Csapó B (2018). Entering school with equal skills? A two-country comparison of early inductive reasoning. Paper presented at the 9th biennial conference of EARLI SIG 1, assessment and evaluation: Assessment & learning analytics; Helsinki.

[ref64] Józsa K. Hricsovinyi J. Szenczi B. (2015). “Számítógép-alapú elsajátítási motiváció kérdőívek validitása és reliabilitása [Validity and reliability of computer-based mastery motivation questionnaires]” in Online diagnosztikus mérések az iskola kezdő szakaszában [Online diagnostic assessments in the beginning phase of schooling]. eds. Csapó B. Zsolnai A. (Budapest: Oktatáskutató és Fejlesztő Intézet), 123–146.

[ref65] Kambeyo L. (2017). The possibilities of assessing students’ scientific inquiry skills abilities using an online instrument: a small-scale study in the Omusati Region, Namibia. Eur. J. Educ. Sci. 4, 1–21. 10.19044/ejes.v4no2a1

[ref66] Kambeyo L. Pásztor A. Korom E. B. Németh M. Csapó B. (2017). Online assessment of scientific reasoning and motivation to learn science: a pilot study in Namibia. Paper presented at the 17th biennial conference for research on learning and instruction; Tampere.

[ref67] Karimova K. Csapó B. (2018). Listening and reading self-concepts in the English and Russian languages. XVIII. Országos Neveléstudományi Konferencia; Budapest.

[ref68] Kárpáti A. Babály B. Simon T. (2015). “A vizuális képességrendszer elemeinek értékelése: térszemlélet és kepi kommunikáció. [Evaluation of the elements of the system of visual skills: spatial representation and pictorial communication]” in Online diagnosztikus mérések az iskola kezdő szakaszában [Online diagnostic assessments in the beginning phase of schooling]. eds. Csapó B. Zsolnai A. (Budapest: Oktatáskutató és Fejlesztő Intézet), 35–69.

[ref69] Kinyó L. (2015). “A társadalmi és állampolgári ismeretek online vizsgálata [Online assessment of civic competencies and knowledge about society]” in Online diagnosztikus mérések az iskola kezdő szakaszában [Online diagnostic assessments in the beginning phase of schooling]. eds. Csapó B. Zsolnai A. (Budapest: Oktatáskutató és Fejlesztő Intézet), 97–121.

[ref70] Klauer K. J. Phye G. D. (2008). Inductive reasoning: a training approach. Rev. Educ. Res. 78, 85–123. 10.3102/0034654307313402

[ref71] Koretz D. (2018). Moving beyond the failure of test-based accountability. Am. Educ. 41, 22–26.

[ref72] Kozma R. (2009). “Transforming education: assessing and teaching 21st century skills. Assessment call to action” in The transition to computer-based assessment. New approaches to skills assessment and implications for large-scale testing. eds. Scheuermann F. Björnsson J. (Luxemburg: Office for Official Publications of the European Communities), 13–23.

[ref73] Kulik J. A. Fletcher J. D. (2016). Effectiveness of intelligent tutoring systems: a meta-analytic review. Rev. Educ. Res. 86, 42–78. 10.3102/0034654315581420

[ref74] Lee M.-K. (2009). “CBAS in Korea: experiences, results and challenges” in The transition to computer-based assessment. New approaches to skills assessment and implications for large-scale testing. eds. Scheuermann F. Björnsson J. (Luxemburg: Office for Official Publications of the European Communities), 194–200.

[ref75] Leighton J. P. Gierl M. J. (eds.) (2007). Cognitive diagnostic assessment for education: Theory and applications. (New York, NY, US: Cambridge University Press).

[ref76] McLachlan C. Fleer M. Edwards S. (2018). Early childhood curriculum: Planning, assessment and implementation: (New York, NY, US: Cambridge University Press).

[ref77] Molnár G. Csapó B. (2018). The efficacy and development of students’ problem-solving strategies during compulsory schooling: logfile analyses. Front. Psychol. 9:302. 10.3389/fpsyg.2018.00302, PMID: 29593606 PMC5855089

[ref78] Molnár G. Csapó B. (2019a). Making the psychological dimension of learning visible: using technology-based assessment to monitor students’ cognitive development. Front. Psychol. 10:1368. 10.3389/fpsyg.2019.01368 31244743 PMC6573803

[ref79] Molnár G. Csapó B. (2019b). A felsőoktatási tanulmányi alkalmasság értékelésére kidolgozott rendszer a Szegedi Tudományegyetemen: elméleti keretek és mérési eredmények [The system developed for the assessment of preparedness for higher educational studies at the University of Szeged: theoretical frameworks and measurement results]. Education (in press).

[ref80] Molnár G. Greiff S. Csapó B. (2013). Inductive reasoning, domain specific and complex problem solving: relations and development. Think. Skills Creat. 9, 35–45. 10.1016/j.tsc.2013.03.002

[ref81] Molnár G. Greiff S. Wüstenberg S. Fischer A. (2017). “Empirical study of computer based assessment of domain-general dynamic problem solving skills” in The nature of problem solving. eds. Csapó B. Funke J. (Paris: OECD), 123–143.

[ref82] Molnár G. Makay G. Ancsin G. (2018). Feladat- és tesztszerkesztés az eDia-rendszerben [Task and test development in the eDia system]. (Szeged: Szegedi Tudományegyetem Oktatáselméleti Kutatócsoport).

[ref83] Molnár G. Papp Z. Makay G. Ancsin G. (2015a). eDia 2.3 Online mérési platform – feladatfelviteli kézikönyv [eDia 2.3 Online assessment platform – task editing manual]. (Szeged: Szegedi Tudományegyetem Oktatáselméleti Kutatócsoport).

[ref84] Molnár G. Pásztor-Kovács A. (2015a). “A problémamegoldó gondolkodás mérése online tesztkörnyezetben [Measurement of problem solving in an online assessment environment]” in Online diagnosztikus mérések az iskola kezdő szakaszában [Online diagnostic assessments in the beginning phase of schooling]. eds. Csapó B. Zsolnai A. (Budapest: Oktatáskutató és Fejlesztő Intézet), 341–366.

[ref85] Molnár G. Tongori Á. Pluhár Z. (2015b). “Az informatikai műveltség online mérése [Online assessment of info-communication literacy]” in Online diagnosztikus mérések az iskola kezdő szakaszában [Online diagnostic assessments in the beginning phase of schooling]. eds. Csapó B. Zsolnai A. (Budapest: Oktatáskutató és Fejlesztő Intézet), 295–317.

[ref86] Mullis I. V. S. Martin M. O. Ruddock G. J. O’ Sullivan C. Y. Arora A. Eberber E. (eds.) (2005). TIMSS 2007 assessment frameworks. (Boston: TIMSS & PIRLS International Study Center, Boston College).

[ref87] Mullis I. V. S. Martin M. O. Smith T. A. Garden R. A. Gregory K. D. Gonzalez E. J. (eds.) (2001). Assessment frameworks and specifications 2003. 2nd Edn. (Boston: International Study Center, Boston College).

[ref88] Munthe E. Rogne M. (2015). Research based teacher education. Teach. Teach. Educ. 46, 17–24. 10.1016/j.tate.2014.10.006

[ref89] Nagy Z. (2015). “A médiahatás vizsgálata általános iskolás tanulók papíralapú és online fogalmazásain [Examination of the paper-based vs. online media effect on composing skills of primary school students]” in Online diagnosztikus mérések az iskola kezdő szakaszában [Online diagnostic assessments in the beginning phase of schooling]. eds. Csapó B. Zsolnai A. (Budapest: Oktatáskutató és Fejlesztő Intézet), 225–244.

[ref90] Nagy L. Korom E. Hódi Á. Németh B. M. (2015). “Az egészségműveltség online mérése [Online assessment of health literacy]” in Online diagnosztikus mérések az iskola kezdő szakaszában [Online diagnostic assessments in the beginning phase of schooling]. eds. Csapó B. Zsolnai A. (Budapest: Oktatáskutató és Fejlesztő Intézet), 147–177.

[ref91] Nikolov M. Csapó B. (2017). Reading abilities in three languages and their relationship to students’ inductive reasoning skills and their parents’ level of education. Paper presented at the conference of the American association for applied linguistics; Atlanta, GA.

[ref92] Nikolov M. Csapó B. (2018). The relationships between 8th graders’ L1 and L2 readings skills, inductive reasoning and socio-economic status in early English and German as a foreign language programs. System 73, 48–57. 10.1016/j.system.2017.11.001

[ref93] OECD (1999). Measuring student knowledge and skills. A new framework for assessment. (Paris: OECD).

[ref94] OECD (2003). The PISA 2003 assessment framework. Mathematics, reading, science and problem solving. (Paris: OECD).

[ref95] OECD (2010). PISA computer-based assessment of student skills in science. (Paris: OECD).

[ref96] OECD (2011). PISA 2009 results: Students on line: Digital technologies and performance (volume VI). (Paris: OECD).

[ref97] OECD (2013). PISA 2012 results: What students know and can do – Student performance in mathematics, reading and science (volume I). (Paris: OECD Publishing).

[ref98] OECD (2014). PISA 2012 results: Creative problem solving. Students’ skills in tackling real-life problems (volume V). (Paris: OECD).

[ref99] OECD (2016). PISA 2015 results (volume I): Excellence and equity in education. (Paris: OECD).

[ref100] OECD (2017). PISA 2015 results (volume V): Collaborative problem solving. (Paris: OECD).

[ref101] Opfer J. E. Nehm R. H. Ha M. (2012). Cognitive foundations for science assessment design: knowing what students know about evolution. J. Res. Sci. Teach. 49, 744–777. 10.1002/tea.21028

[ref102] Pachler N. Daly C. Mor Y. Mellar H. (2010). Formative e-assessment: practitioner cases. Comput. Educ. 54, 715–721. 10.1016/j.compedu.2009.09.032

[ref103] Pásztor A. Kupiainen S. Hotulainen R. Molnár G. Csapó B (2018). Comparing Finnish and Hungarian fourth grade students’ inductive reasoning skills. Paper presented at the 9th biennial conference of EARLI SIG 1, assessment and evaluation: Assessment & learning Analytics; Helsinki.

[ref104] Pásztor A. Molnár G. Csapó B. (2015). Technology-based assessment of creativity in educational context: the case of divergent thinking and its relation to mathematical achievement. Think. Skills Creat. 18, 32–42. 10.1016/j.tsc.2015.05.004

[ref105] Pásztor-Kovács A. Pásztor A. Molnár G. (2018). Kollaboratív problémamegoldó képességet vizsgáló dinamikus teszt fejlesztése [Development of an online interactive instrument for assessing collaborative problem solving]. Magyar Pedagógia 118, 73–102. 10.17670/MPed.2018.1.73

[ref106] Pellegrino J. W. Chudowsky N. Glaser R. (eds.) (2001). Knowing what students know: The science and design of educational assessment. (Washington, DC: National Academies Press).

[ref107] Pellegrino J. W. Quellmalz E. S. (2010). Perspectives on the integration of technology and assessment. J. Res. Technol. Educ. 43, 119–134. 10.1080/15391523.2010.10782565

[ref108] Perie M. Marion S. Gong B. (2009). Moving towards a comprehensive assessment system: a framework for considering interim assessments. Educ. Meas. Issues Pract. 28, 5–13. 10.1111/j.1745-3992.2009.00149.x

[ref109] Plichart P. Jadoul R. Vandenabeele L. Latour T. (2004). TAO, A collective distributed computer-based assessment framework built on semantic web standards. Proceedings of the international conference on Advances in intelligent Systems – Theory and application AISTA2004, in cooperation with IEEE computer society; 2004 Nov 15–18; Luxembourg. Luxembourg.

[ref110] Ragchaa J. (2017). Mongolian students’ learning strategies in mastering English receptive skills. Paper presented at the 5th RSEP social sciences conference; Barcelona.

[ref111] Ryan R. M. Deci E. L. (2000a). Intrinsic and extrinsic motivations: classic definitions and new directions. Contemp. Educ. Psychol. 25, 54–67. 10.1006/ceps.1999.1020 10620381

[ref112] Ryan R. M. Deci E. L. (2000b). Self-determination theory and the facilitation of intrinsic motivation, social development, and well-being. Am. Psychol. 55, 68–78. 10.1037/0003-066X.55.1.68 11392867

[ref113] Saeki E. Segool N. Pendergast L. von der Embse N. (2018). The influence of test-based accountability policies on early elementary teachers: school climate, environmental stress, and teacher stress. Psychol. Sch. 55, 391–403. 10.1002/pits.22112

[ref114] Scandura J. M. (2017). Contrasting fundamental assumptions in adaptive learning and modeling human tutors with TutorIT. Technol. Instr. Cogn. Learn. 10, 259–265.

[ref115] Scardamalia M. Bransford J. Kozma B. Quellmalz E. (2012). “New assessments and environments for knowledge building” in Assessment and teaching of 21st century skills. eds. Griffin P. McGaw B. Care E. (New York: Springer), 231–300.

[ref116] Scheuermann F. Björnsson J. (eds.) (2009). The transition to computer-based assessment. New approaches to skills assessment and implications for large-scale testing. (Luxemburg: Office for Official Publications of the European Communities).

[ref117] Scheuermann F. Guimarães Pereira A. (eds.) (2008). Towards a research agenda on computer-based assessment: Challenges and needs for European educational measurement. (Ispra: European Commission Joint Research Centre).

[ref118] Sheard M. K. Chambers B. (2014). A case of technology-enhanced formative assessment and achievement in primary grammar: how is quality assurance of formative assessment assured? Stud. Educ. Eval. 43, 14–23. 10.1016/j.stueduc.2014.02.001

[ref119] Sindelar N. W. (2010). Assessment-powered teaching. (Thousand Oaks, CA: Corwin Press).

[ref120] Slavin R. E. (2002). Evidence-based education policies: transforming educational practice and research. Educ. Res. 31, 15–21. 10.3102/0013189X031007015

[ref500] Sørensen H. Andersen A. M. (2009). “How did Danish students solve the PSA CBAS items?” in The transition to computer-based assessment. New approaches to skills assessment and implications for large-scale testing. eds. Scheuermann F. Björnsson J. (Luxemburg: Office for Official Publications of the European Communities), 201–208.

[ref121] Tongori Á. (2018). Measuring ICT literacy among grade 5–11 students: Confidence in accessing information. Unpublished doctoral dissertation. Szeged: University of Szeged.

[ref122] Tóth E. (2011). Pedagógusok nézetei a tanulói teljesítménymérésekről [Teachers’ views of learner assessment programmes]. Magyar Pedagógia 111, 225–249.

[ref123] Tóth E. (2015). “A gazdasági műveltség diagnosztikus mérésének lehetőségei online környezetben [The possibilities of assessing financial literacy in an online environment]” in Online diagnosztikus mérések az iskola kezdő szakaszában [Online diagnostic assessments in the beginning phase of schooling]. eds. Csapó B. Zsolnai A. (Budapest: Oktatáskutató és Fejlesztő Intézet), 269–293.

[ref124] Tóth K. Rölke H. Goldhammer F. Barkow I. (2017). “Educational process mining: new possibilities for understanding students’ problem-solving skills” in The nature of problem solving. Using research to inspire 21st century learning. eds. Csapó B. Funke J. (Paris: OECD), 193–209).

[ref125] Vainikainen M. P. Hautamäki J. Hotulainen R. Kupiainen S. (2015). General and specific thinking skills and schooling: preparing the mind to new learning. Think. Skills Creat. 18, 53–64. 10.1016/j.tsc.2015.04.006

[ref126] Vígh T. Sominé Hrebik O. Thékes I. Vidákovich T. (2015). “Fiatal nyelvtanulók német és angol alapszókincsének diagnosztikus vizsgálata [Diagnostic assessment of young learners’ basic English and German vocabulary]” in Online diagnosztikus mérések az iskola kezdő szakaszában [Online diagnostic assessments in the beginning phase of schooling]. eds. Csapó B. Zsolnai A. (Budapest: Oktatáskutató és Fejlesztő Intézet), 13–33.

[ref127] Westbury I. Hansén S. E. Kansanen P. Björkvist O. (2005). Teacher education for research-based practice in expanded roles: Finland’s experience. Scand. J. Educ. Res. 49, 475–485. 10.1080/00313830500267937

[ref128] Wilson M. Bejar I. Scalise K. Templin J. Wiliam D. Irribarra T. (2012). “Perspectives on methodological issues” in Assessment and teaching of 21st century skills. eds. Griffin P. McGaw B. Care E. (New York: Springer), 67–141.

[ref129] Wu H. Molnár G. (2018a). Interactive problem solving: assessment and relations to combinatorial and inductive reasoning. J. Psychol. Educ. Res. 26, 90–105.

[ref130] Wu H. Molnár G. (2018b). Computer-based assessment of Chinese students’ component skills of problem solving: a pilot study. Int. J. Inf. Educ. Technol. 8, 381–356. 10.18178/ijiet.2018.8.5.1067

[ref131] Zsolnai A. Kasik L. (2015). “Az együttműködő viselkedés és az alapérzelem-felismerés online vizsgálata [Online assessment of the recognition of cooperative behavior and basic emotions]” in Online diagnosztikus mérések az iskola kezdő szakaszában [Online diagnostic assessments in the beginning phase of schooling]. eds. Csapó B. Zsolnai A. (Budapest: Oktatáskutató és Fejlesztő Intézet), 71–95.

